# A Microfluidic Platform for Cavitation-Enhanced Drug Delivery

**DOI:** 10.3390/mi12060658

**Published:** 2021-06-03

**Authors:** Giulia Grisanti, Davide Caprini, Giorgia Sinibaldi, Chiara Scognamiglio, Giulia Silvani, Giovanna Peruzzi, Carlo Massimo Casciola

**Affiliations:** 1 Department of Mechanical and Aerospace Engineering, Sapienza University of Rome, Via Eudossiana 18, 00186 Roma, Italy; giulia.grisanti@uniroma1.it (G.G.); Giorgia.sinibaldi@uniroma1.it (G.S.); giulia.silvani@uts.edu.au (G.S.); 2 Center for Life Nano- & Neuro-Science, Fondazione Istituto Italiano di Tecnologia (IIT), Via Regina Elena 291, 00161 Roma, Italy; davide.caprini@iit.it (D.C.); chiara.scognamiglio@iit.it (C.S.); 3 School of Biomedical Engineering, Faculty of Engineering & Information Technology, University of Technology Sydney, Ultimo, NSW 2007, Australia

**Keywords:** endothelium permeabilization, drug delivery, microfluidics

## Abstract

An endothelial-lined blood vessel model is obtained in a PDMS (Polydimethylsiloxane) microfluidic system, where vascular endothelial cells are grown under physiological shear stress, allowing -like maturation. This experimental model is employed for enhanced drug delivery studies, aimed at characterising the increase in endothelial permeability upon microbubble-enhanced ultrasound-induced (USMB) cavitation. We developed a multi-step protocol to couple the optical and the acoustic set-ups, thanks to a 3D-printed insonation chamber, provided with direct optical access and a support for the US transducer. Cavitation-induced interendothelial gap opening is then analysed using a customised code that quantifies gap area and the relative statistics. We show that exposure to US in presence of microbubbles significantly increases endothelial permeability and that tissue integrity completely recovers within 45 min upon insonation. This protocol, along with the versatility of the microfluidic platform, allows to quantitatively characterise cavitation-induced events for its potential employment in clinics.

## 1. Introduction

To develop efficacious therapeutic approaches, the elaboration of effective delivery strategies has proven to be a challenging step for researchers. Throughout the 19th century, starting from Paul Ehrlich’s concept of “magic bullet” [1], pharmaceutical studies brought the realisation of different generations of controlled and targeted drug delivery systems, at the macro-, micro- and nanoscopic scale [2]. The rationale of maximising drug concentration at the site of interest lies in the consequent reduction of the administered dose needed to obtain the therapeutic activity, preventing harmful off-target effects [3]. In this respect, various strategies have been adopted, such as the drug-target recognition at the molecular level and the employment of transporting units, the so-called “drug carriers”, interacting with specific components of the pharmaceutical target. The choice of the optimal option is affected by several factors, including target biological complexity and administration route [3]. In particular, several hurdles arise from drug administration into the cardiocirculatory system, involving its delivery to the site of interest and its stability in the blood stream. In this context, drug carriers are essential and need to fulfill four requirements: *retain* the drug until needed, *evade* the immune system, *target* the tissue of interest and *release* the drug at the target site [4]. One of the main obstacles to the effectiveness of drug delivery strategies is represented by biological barriers, i.e., biological structures that guarantee organ integrity and homeostasis by separating the internal from the external environment in organs, tissues and cells. By allowing the selective transfer of specific molecules across them, biological barriers protect cells and tissues, preventing the passage of potentially harmful or toxic molecules, like drugs [5,6].

The endothelium is the biological barrier par excellence. It is composed by endothelial cells (ECs) compacted in a monolayer lining the lumen of blood and lymphatic vessels. It ensures the homeostasis of the cardiocirculatory system and acts as a semipermeable, size-selective barrier, regulating the exchanges of gas and specific nutrients between the blood stream and the neighbouring tissues, contemporarily blocking all other molecules, including drugs [6,7,8]. Such crucial functions are deeply connected to the endothelial structure and to the presence of different interendothelial junction complexes: adherens, tight and gap junctions [8,9]. Adherens junctions (AJs) are ubiquitous in the vascular tree and regulate endothelial barrier integrity and permeability, as well as several other ECs’ physiological activities [8,9,10]. They are mainly constituted by the adhesion protein vascular endothelial (VE)-cadherin, which mediates cell–cell contacts at the extracellular side [10,11], whereas it interacts with signalling and scaffolding proteins at the intracellular side, where it is also connected to the cell actin cytoskeleton [10,12]. This implies that the remodelling of the actin cytoskeleton also affects VE-cadherin organisation in different morphological patterns, ranging from linear to interrupted, reticular or plaque-like structures [13]. For their dynamic organisation, these junctions can easily reorganise their molecular configuration and open interendothelial passages in response to different mechanical stimuli [7,10].

Given the key role of the endothelium in affecting the efficiency of drug delivery, its features and permeability have been subject of thorough investigation. Recent studies have investigated the biological barrier in artificial systems reproducing physiological conditions, employing the ground-breaking technologies offered by microfluidics. It entails the manipulation of small amounts of fluids (nano- or microlitres) within micrometre-sized devices, usually made of polydimethylsiloxane (PDMS) and comprising customisable networks of microchannels [14]. Cell cultures can be grown within these platforms with biologically relevant features, reproducing the complexity of an organ in terms of microenvironment and 3D organisation, even including physiological levels of pressure, shear stress and dynamical mechanical stimuli [15,16]. The thorough characterisation of the physical parameters related to device geometry [17,18] allows system optimisation to favour cell growth and molecular delivery. For these reasons, these platforms allow to perform molecular, pharmaceutical, diagnostic or high-throughput analyses in controlled and reproducible conditions [18,19,20,21]. They also contribute to overcome the ethical issues related to models [22] and have the potential to be employed in the early stages of clinical trials [23]. Therefore, microfluidic platforms offer a valid alternative to traditional 2D culture models (flasks or Transwell), which are inadequate when it comes to structural and 3D organisation. Thanks to the advantages offered by microfluidics, several endothelial and blood–brain barrier (BBB) models have been created in order to characterise the properties of these barriers in physiological and pathological conditions, as well as for pharmaceutical and therapeutic applications [24,25,26,27,28,29,30,31,32].

Microfluidic platforms allowed to highlight the importance of physiological shear stress (1–12 dyn cm${}^{-2}$ in microvasculature) for the formation of a mature vascular lumen, favouring streamwise cell elongation and stabilising junction proteins [25,33,34,35]. Upon endothelial maturation, barrier permeability can be characterised through different approaches [36]. Taking advantage of the presence of a blood vessel-like structure, known-molecular-weight fluorescent tracers (e.g., Alexa-Fluor-labeled bovine serum albumin (BSA) [27,37,38] or fluorescent dextran [25,39]) can be injected in the vascular microchannels, and their passage through the endothelial barrier is monitored over time, to extract the permeability coefficient from the fluorescence intensity profile [40]. Another common technique is the transendothelial electrical resistance (TEER), which consists in the quantitative measurement of the resistance of the endothelial monolayer. It is carried out by placing two electrodes, one on each side of the biological barrier, and measuring the momentary resistance in response to an applied potential [36,41,42].

Several approaches have been developed to increase endothelial permeability and maximise drug delivery. Effective strategies include the direct application of the drug at the target site, the use of target-specific carriers, the exploitation of leaking vessels (as in case of cancer) to obtain passive accumulation of the drug, and physical/magnetic targeting of the tissue [3].

Another advanced approach that has lately generated growing interest is based on the physical phenomenon of cavitation, occurring upon the exposure of gas microbubbles (MBs) to ultrasound (US). These two elements find wide application in clinics. Indeed, MBs (∼1–10 μm in diameter) are made of a heavy-molecular-weight inert gas core enclosed in a stabilising lipid shell and are routinely used as ultrasound contrast agents (UCA) in diagnostics [43,44,45], while US is largely employed as a diagnostic and therapeutic tool [44]. Cavitation and its implications in enhanced drug delivery have been widely discussed in the literature [44,46,47,48]. Briefly, MBs exposed to a beam of (plane or focused) US respond to the oscillatory acoustic pressure by undergoing expansion/compression cycles, with the oscillation amplitude depending on US intensity, thus on the acoustic pressure, and being influenced by the surface tension, the inertia and the viscosity of the surrounding fluid [49,50]. Two different kinds of cavitation can be distinguished [48,51,52]. Stable cavitation occurs at low acoustic pressure (US intensity 0.3–3 W cm${}^{-2}$), which causes MBs to linearly oscillate in a stable motion, below a critical size [53], displacing the surrounding fluid and thus generating steady microstreamings. Conversely, for US intensity greater than 3 W cm${}^{-2}$, inertial cavitation involves the non-linear and unstable expansion of MBs up to a critical radius, at which they violently collapse asymmetrically and successively re-expand back. This causes the compressed gas to warm up and provokes secondary mechanical phenomena, such as the emission of shock waves and liquid jets in the surrounding liquid [54,55].

These phenomena underlie several bioeffects. USMB-mediated cavitation alters the cell membrane integrity, due to the mechanical stress exerted on cells by both MBs and fluid microstreaming. Membrane equilibrium is destabilised, provoking the formation of pores within the lipid bilayer, proportionally with acoustic pressure and MBs oscillation amplitude [56,57,58,59]. Moreover, alteration of cell radical oxygen species (ROS) homeostasis and increase of Ca${}^{2+}$ intracellular levels have been recorded upon cavitation [60]. When MBs are injected into the lumen of a blood vessel and exposed to US excitation, cytoskeletal rearrangements take place and the increase in F-actin stress fibres generate tension within the cells, affecting interendothelial junction complexes linked to the actin cytoskeleton. As a result, VE-cadherin molecules loose contacts and the junctions are disrupted, altering the integrity of the whole endothelium. This leads to tissue permeabilisation, which has been demonstrated to be a temporary event, as the restoration of intercellular contacts has been observed in 2D cell cultures within 30 min after US irradiation [60,61].

These effects can be induced by US alone, but are intensified by pre-existing MBs, which act as local acoustic amplifiers [50,61,62]. This localises the bioeffects to the vasculature and reduces adverse consequences, such as bleeding, endothelial leaking or ischemic apoptotic areas [63,64,65]. For these reasons, UCAs have been proposed for therapeutic applications and are currently employed in several enhanced drug delivery strategies, including gene therapy, cardiovascular and cerebral drug delivery [44,66,67,68,69,70,71].

In this scenario, our group recently employed a blood vessel-on-chip model, originally developed in [25], to characterise cavitation-enhanced endothelial permeability [35,72]. The purpose of the present paper is to describe details concerning the set-up, its design and fabrication procedure, together with providing examples of application of the device to cavitation-enhanced endothelial layer permeability through the formation of interendothelial gaps. The interest is focused on the acoustic and optical components of the system, as well as on the image analysis procedure, through a self-customised MATLAB code used to identify and extract interendothelial gaps and extrapolate information on USMB-mediated cavitation effects.

The microfluidic device comprised a central compartment interconnected with two surrounding and independent vascular microchannels. Human umbilical vein endothelial cells (HUVECs) were grown under physiologically relevant flow conditions, reproducing the shear stress exerted by the blood stream on microvasculature. Upon endothelial maturation, cells lined the vascular lumen showing well-defined VE-cadherin patterns. Lipid-coated MBs (SonoVue^®^) were injected into one of the two vascular channels and the system was irradiated with low intensity US at 1 MHz for 30s at two different acoustic pressures: 0.4 MPa and 0.72 MPa. US exposure caused stable cavitation to occur, being the threshold for inertial cavitation at 0.86 MPa for these experimental conditions (low fluid speed of 0.83 mm s${}^{-1}$) [73]. Results confirmed significant endothelial permeabilisation at the acoustic pressure of 0.72 MPa, through the opening of intercellular gaps, caused by local disruption of cell–cell contacts due to VE-cadherin rearrangement. This effect, already considerable when the endothelium is treated with US alone, is intensified by the presence of MBs, with an increase of the total gap area of 360% with respect to the control condition (i.e., non treated mature endothelium). Moreover, when the endothelium was exposed to physiological levels of shear stress (10 dyn cm${}^{-2}$) upon US irradiation, total recovery of tissue integrity was registered after 45 min, with the closure of the gaps formed by cavitation [35].

## 2. Materials and Methods

### 2.1. Device Fabrication

**Figure 1:**
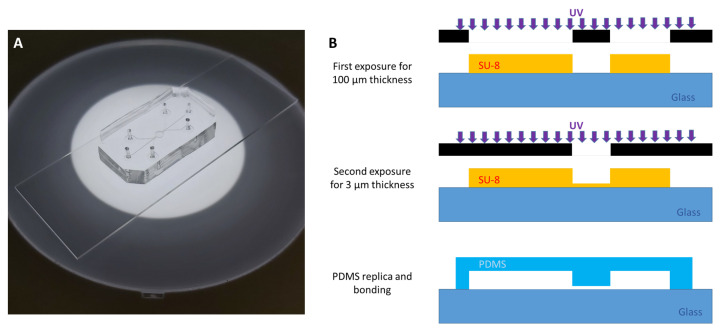
(**A**) Picture of the customised PDMS device. (**B**) Main steps of the photolithography process to realise the mould for the microfluidic device (details in Section 2.1).

In order to choose the most suitable commercial device, tests were conducted on self-fabricated devices based on the SynVivo geometry, described in [25] and shown in Figure 1A. Different chips were developed with different spacing distances between the pores that connect the tissue compartment and the microchannels. These kinds of geometries are characterised by different thicknesses in the channels of the microfluidic structure. Therefore, they require a special multilayer manufacturing process [74].

The fabrication of two-layer chips using PDMS elastomer is based on soft-lithography [75]. The procedure starts with the fabrication of the cured photoresist moulds via the classic photolithography process, with the main steps depicted in Figure 1B. The substrate used for photolithography is borosilicate-glass, and the resist to define the structures is SU-8 (epoxy negative). The multilayer moulds are fabricated using the typical process of SU-8 repeated twice in consecutive steps. The first exposure is made with a structure for the tissue compartment and the vascular channels, which are 100 μm thick, while the second exposure, after the first soft-baking, allows to obtain the part of the channels with a thickness of 3 μm. This double step allows the multilayer fabrication. Fabrication of the devices is obtained via the standard PDMS casting and replica process that is required in single-layer soft lithography.

### 2.2. Cell Culture and of the Vasculature on Chip

HUVECs and their culture medium, the endothelial basal medium-2 (EBM-2) supplemented with endothelial growth medium (EGM-2) BulletKit, were purchased from Lonza (Walkersville, MD, USA). Cells were used up to the 5th passage.

For a detailed description of the cell culture and seeding protocols, see [25,35,76]. Briefly, HUVECs were cultured in tissue culture flasks at 37 ${}^{\circ }$C and 5% CO${}_{2}$ until they reached 80–90% confluence, when they were detached from the flask surface and resuspended at the final average concentration of $10^{8}$ cells mL${}^{-1}$. At this concentration, cells were seeded in the vascular channels of the microfluidic device, which had been previously functionalised with fibronectin (200 μg mL${}^{-1}$) (Sigma-Aldrich, St Louis, MO, USA), incubated for 2 h at 37 ${}^{\circ }$C and 5% CO${}_{2}$. HUVECs were injected in the device microchannels with the aid of a programmable syringe pump (PhD ULTRA Syringe Pump, Harvard Apparatus, Holliston, MA, USA). The initial cell density was optimised to 60–70%, so as to guarantee the formation of a mature endothelium lining the device microchannels. Then, they were allowed to adhere to the PDMS walls in static conditions at 37 ${}^{\circ }$C and 5% CO${}_{2}$. After 4-h incubation, the inlets of the vascular channels were connected to growth medium reservoirs through Tygon tubes (Saint Gobain PPL Corp., Solon, OH, USA), whereas the Tygon tubes of the channel outlets were attached to syringes placed on a double syringe pump, in order to pull the medium into the channels. Flow perfusion reached the rate of 0.5 μL min${}^{-1}$ over the first 24 h, then the medium was refreshed and the flow rate was ramped up to 25 μL min${}^{-1}$ (exerting a shear stress equal to 10 dyn cm${}^{-2}$, respectively), which induced ECs to elongate streamwise. After 2 days of flow perfusion, the endothelial layer reached full junction maturation. In this condition, ECs constitute a compact monolayer of cells at 90–95% confluence, comprising exclusively viable cells, since dead ones detach from the vessel walls and are washed out by the flow.

Endothelial maturation can be evaluated by the observation of VE-cadherin pattern through immunofluorescence (IF) assay. The obtaining of a compact vascular barrier is crucial for the successive steps of the USMB-mediated cavitation experiments, which were performed immediately after. However, we have indications that the phenotypical features of the formed barrier can be maintained for at least two days.

### 2.3. Immunofluorescence Staining

All the steps of the IF protocol were carried out at room temperature (RT) conditions. Initially, samples were fixed with paraformaldehyde (PFA), for 15 min in static conditions, and then rinsed with PBS. Then, ECs were permeabilised with 0.2% Triton X-100 (Sigma-Aldrich, MO, USA) for 5 min. To monitor cell–cell junctional complexes, VE-cadherin was stained by incubating HUVECs with 5 μg mL${}^{-1}$ VE-cadherin mouse monoclonal antibody (Thermo Fisher Scientific, Weltham, MA, USA) in 3% BSA for 1 h in the dark, perfused at 0.5 μL min${}^{-1}$. Then, AlexaFluor647 conjugate-Goat anti-mouse IgG (H+L) secondary antibody (2 μg mL${}^{-1}$) (Thermo Fisher Scientific) was perfused for 1 h at 0.5 μL min${}^{-1}$ and antibody excess was washed with PBS afterwads. Nuclei were stained with DAPI (Thermo Fisher Scientific) for 5 min in static conditions and then rinsed with PBS.

### 2.4. Insonation Chamber Design

In order to carry out USMB-induced cavitation experiments on the vasculature-on-chip system, an insonation chamber was designed and developed to integrate the US chain with direct visualisation of the events through brightfield microscopy and time-lapse recordings.

**Figure 2:**
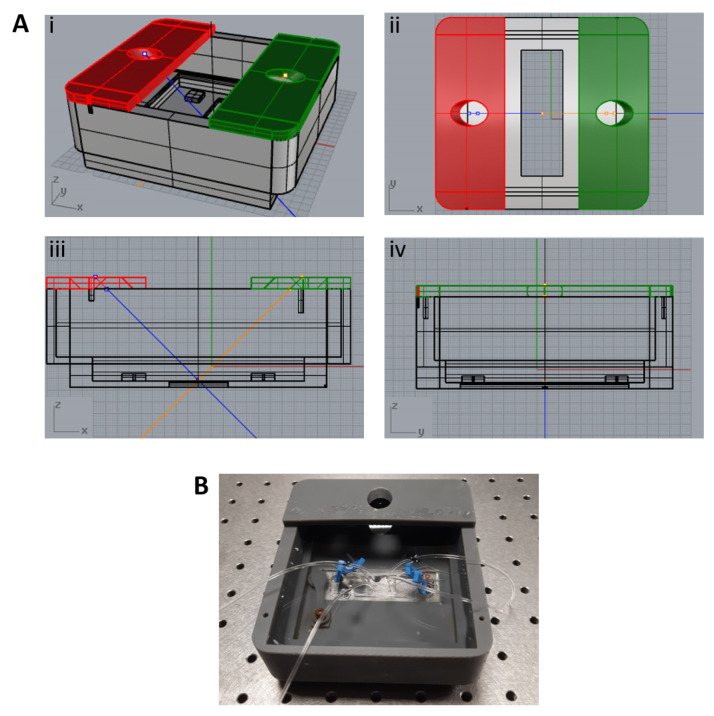
(**A**) Technical drawing of the insonation chamber prototype: (i) overview, (ii) top view, (iii) and (iv) lateral views. (**B**) Picture of the 3D-printed insonation chamber.

**Figure 3:**
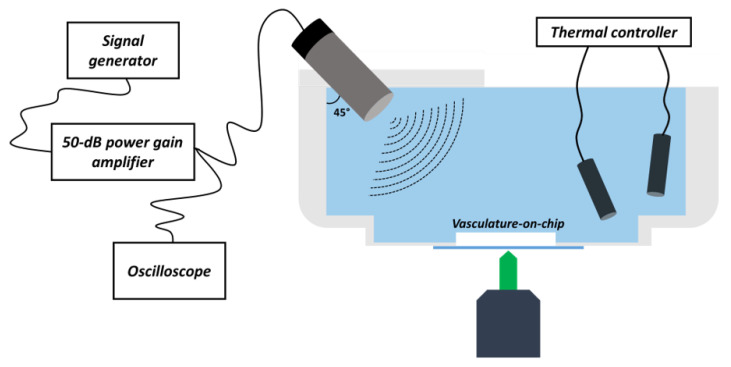
Sketch of the acoustic and optical set-ups of the USMB-induced cavitation experiment showing the integration of the acoustic and optical components (see the Supplementary Material for additional information). The chamber was designed to host the microfluidic device at its bottom, guaranteeing optical access to the microscope objectives. In order to allow US waves to correctly propagate from the $45^{\circ }$-inclined piezo located at the top, the chamber was filled with deionised water. Water temperature was constantly monitored and maintained at 37 ${}^{\circ }$C with the aid of a thermal controller. The piezo was connected to the US chain, comprising signal generator, 50-dB amplifier, and oscilloscope to monitor the US bursts.

The design was made through computer-aided design (CAD) using the Rhinoceros software. The insonation chamber is realised through 3D printing and is made of an inert plastic resin, which must guarantee structural strength and total impermeability. Figure 2 shows a picture of the chamber and relative technical drawing. The chamber was tailor-made for the stage of a Olympus spinning disk confocal fluorescent microscope, as in Figure 3. The external chamber size was $13\times 11.5\times 4.5\;{\mathrm{cm}}^{3}$; while the internal chamber measured $11.5\times 10\times 3.5\;{\mathrm{cm}}^{3}$. In accordance with these technical specifications, the chamber could host a volume of approximately 0.4 l of deionised water which matches the impedance at the PDMS interface.

The microfluidic platform is placed at the bottom of the support, where a notch sealed with transparent plexiglass guarantees direct visualisation of the blood vessel model. The total thickness of the plexiglass and of the device microscope glass lied within the working distance of microscope objectives typically used (4×, 10×, 20×). The design also included a pin to secure the microfluidic device, with a dual purpose: to avoid device displacement once the chamber is filled with deionised water and to maintain it wholly in contact with the surface of the plexiglass, in order to prevent air infiltration, which could hamper microscope observation.

In order to integrate this system with the acoustic chain, a support for the piezo transducer was designed on the top of the chamber, yet leaving an appropriate surface free. This is needed to allow the transmitted microscope light to pass through and reach the microfluidic device, as well as to let enough room for the inlet and outlet Tygon tubes to exit from the chamber and connect the vasculature with the syringe pump. The transducer support kept it at $45^{\circ }$ inclination, at a distance of 35 mm from the microfluidic platform, as explained more in detail in the Supplementary Material.

### 2.5. Acoustic Set-Up

As discussed in Section 1, cavitation was used as a therapeutic strategy to enhance endothelial permeability. Preliminarily to the experiments carried out to evaluate the opening of interendothelial gaps, system characterisation and setting-up of the acoustic and insonation protocol were finalised. The outline of the acoustic chain and its integration with the optical set-up through the insonation chamber is shown in Figure 3.

US bursts were generated through the acoustic chain: electrical signals were emitted in bursts by a signal generator and transmitted to a 50-dB power gain amplifier. The subsequently amplified signals were thus sent to a planar, single element, 1-MHz-centre-frequency transducer (diameter: 12.7 mm), which converted them in sine-wave US beams. All the process involving the generation and amplification of the waves were constantly monitored with the aid of an oscilloscope, which reads the amplifier output.

Several US-related parameters can influence MBs behaviour, including the excitation frequency, acoustic beam pulse length (PL), pulse repetition frequency (PRF) and duty cycles (DC), as well as the Peak Negative Pressure (PNP), i.e., pressure amplitude. However, other factors related to MBs and to the surrounding fluid are involved too, such as the surface tension as well as fluid inertia and viscosity [49,50].

For this investigation, the following parameters were chosen: 1 MHz central frequency, 500 cycles repeated every 50 ms, 0.1% DC, corresponding to 500 μs pulse duration (PD), and 20 Hz PRF. The total duration of insonation was 30 s (corresponding to 600 pulses). Two different acoustic pressures were considered, namely the PNP of 0.4 MPa, corresponding to $5\;W\;{\mathrm{cm}}^{-2}$ intensity and 80 mV transducer-driving voltage, and the PNP of 0.72 MPa, corresponding to $17\;W\;{\mathrm{cm}}^{-2}$ intensity and 140 mV transducer-driving voltage. Such levels of pressure, along with the parameters of the neighbouring liquid (fluid speed of 0.83 mm s${}^{-1}$), ensured the occurrence of stable cavitation, as explained in Section 1.

### 2.6. Microbubbles

SonoVue^®^ (Bracco Research, Geneva, Switzerland) MBs were chosen to carry out the present investigation.

Their dynamics were characterised in [77] using an ultrafast camera. That work also proposes an improvement over the minimal model provided by the celebrated Rayleigh–Plesset equation for the time-dependent gaseous bubble radius R(t) [78]

(1) $$\frac{p_{B}\left(t\right)-p_{L}^{\infty }}{\rho_{L}\left(t\right)}\;=\;R\ddot{R}+\frac{3}{2}{\dot{R}}^{2}+\frac{4\mu_{L}}{\rho_{L}}\frac{\dot{R}}{R}+\frac{2\sigma }{\rho_{L}R},$$

where $\rho_{L}$ is the liquid density, $\mu_{L}$ the dynamic viscosity, $p_{B}\left(t\right)$ is the pressure inside the bubble and $p_{L}^{\infty }\left(t\right)$ is the time-dependent acoustic pressure, with σ as the liquid/gas surface tension. The extended model [77] accounts for the phospholipid monolayer that coats the bubble and affects the dynamics in a highly non-trivial way. When the bubble surface shrinks below a threshold (buckling limit area, $A_{buckling}$, determined by the number of phospholipid molecules and their head-group area [79]), the surface tension vanishes. Above that limit, the bubble acquires an elastic behaviour with modulus χ. After a critical radius, the coating breaks, exposing the gas core to the water. The effective surface tension is then approximated as [77]

(2) $$\begin{array}{c} \sigma \left(R\right)=\left\{\begin{array}{cc} 0 & R\leq R_{buckling}=\sqrt{A_{buckling}/\pi }\\ \chi \left(\frac{R^{2}}{R_{buckling}^{2}}-1\right) & R_{buckling}\leq R\leq R_{break-up}\\ \sigma_{water} & \mathrm{after}\;break-up \end{array}\right.\;. \end{array}$$

In the present experiments, the MBs suspension was reconstituted in 5mL solution of 0.2%NaCl ($2\times 10^{8}÷5\times 10^{8}$ MBs mL${}^{-1}$), according to manufacturer’s instructions. This preparation was then diluted to the concentration of $2\times 10^{7}÷5\times 10^{7}\;{\mathrm{mL}}^{-1}$ in culture medium (enriched with 2.5% HEPES buffer solution) to obtain 1:1 cell-to-bubble ratio [80,81].

### 2.7. USMB-Mediated Cavitation Experiments

Once the endothelium reached complete maturation under physiological-like flow conditions, the vasculature-on-chip model was employed to investigate the increase of endothelial permeability upon USMB-mediated cavitation.

The system was mounted onto the microscope stage. The vascular channels were continuously perfused with culture medium enriched with 2.5% HEPES at the rate of 25 μL min${}^{-1}$. Initially, ECs were let adapt to the new conditions for 30 min with a constant temperature monitoring made by a PID (proportional integral derivative) thermal controller, which maintained the water temperature at 37 ${}^{\circ }$C (thermalisation step). During this phase, cells were looked over through brightfield time-lapse recordings. Then, SonoVue^®^ MB suspension was injected into one of the two vascular channels.

It should be stressed that a capillary vessel has a typical diameter in the range 5–20 μm with a physiological shear stress in the range 1–12 $\;\mathrm{dyn}\;{\mathrm{cm}}^{-2}$. A typical shear stress of $10\;\mathrm{dyn}\;{\mathrm{cm}}^{-2}$ in a capillary with 10μm diameter corresponds to an average velocity $V_{c}=1.25\;\mathrm{mm}\;s^{-1}$ [82]. The same shear stress is obtained in the device (cross section $S=100\times 200\;\mu m^{2}$) at a flow rate of $25\;\mu L\;\min^{-1}$, corresponding to a velocity $V_{d}=20\;\mathrm{mm}\;s^{-1}$. This speed is more than ten times larger than in the actual blood vessel. As a consequence, bubbles flow too fast and remain over the endothelium for too short a time to produce the same physiological effect. There are then two incompatible requirements: same shear stress and similar bubble transit time. The strategy to comply with these restrictions is to always keep the device under the flow rate of 25 $\;\mu L\;\min^{-1}$ except for the short insonation period, when the flow rate is temporarily reduced to $1÷1.5\;\mu L\;\min^{-1}$ to slow down the velocity to the same values as in the actual capillary.

Hence, when MBs reached a suitable concentration in the microchannel, the flow rate was transiently reduced to $1\;\mu {L\;\min}^{-1}$ to ensure a sufficient MBs residence time over the vasculature. At this point, US exposure was started. The MBs injection in one of the two vascular channels featured by the device allows the simultaneous investigation of two different conditions: US exposure in presence of MBs (in the channel where SonoVue^®^ were injected) and US alone (in the other channel). This step was monitored through brightfield time-lapse recording. In parallel, control experiments were run with identically prepared samples with no insonation.

After 30 s, US was stopped and cells were fixed with PFA within 4 min from the end of the insonation, in order to evaluate the effect of cavitation. On the contrary, when assessing endothelium integrity recovery, the vasculature was re-exposed to fluid flow at 25 μL min${}^{-1}$ for 45 min and finally cells were fixed to evaluate the endothelial conditions.

### 2.8. Optical Set-Up and IF Image Acquisition

An inverted Olympus iX73 equipped with X-light V1 spinning disk head (Crestoptics, Roma, IT) and Lumencor Spectra X LED illumination was employed to perform sample imaging with Olympus 20× air objective (NA = 0.45) and MetaMorph software (Molecular Devices, San Jose, CA, USA).

Phase contrast imaging was used to monitor the vascular channels in real-time during the overall duration of cavitation experiments, in order to guarantee the integrity of the endothelial barrier and follow MBs dynamics. For this purpose, time lapse recordings were performed using an Evolve EMCCD camera (Photometrics, Tucson, AZ, USA), with different acquisition parameters: 1 fps at 300 ms exposure time during thermalisation and 10 fps at 10 ms exposure time during insonation in the presence of MBs.

**Figure 4:**
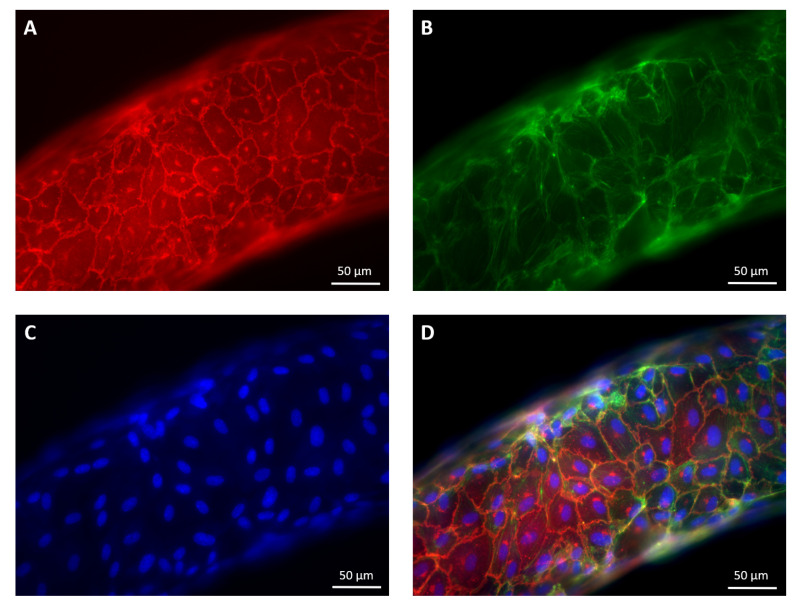
Examples of confocal fluorescence tiles captured for the same portion of the vascular channel with different colour channels. (**A**) VE-cadherin. (**B**) Actin. (**C**) Cell nuclei. (**D**) Merging of the fluorescence images.

The fixed samples were stained for VE-cadherin, actin and DAPI, Figure 4. IF images were acquired using a CoolSNAP MYO CCD camera (Photometrics, AZ, USA) with the 20× objective, that provides sufficient resolution to measure the opened interendothelial gaps. At this magnification, the whole specimen does not fit the microscope field of view. Hence, images of different portions of the endothelium were stitched to assemble the whole vascular channels. The subdivision in tiles allowed sufficient overlap for the reconstruction of the entire image.

Confocal Z-stack images of the endothelium stained for VE-cadherin and cell nuclei were also acquired at 20× magnification to obtain a fully three dimensional view of the channel through the Imaris software (Oxford Instruments, Abington, UK).

### 2.9. Interendothelial Gap Analysis

The acquired images were processed through a multi-step procedure that entails the initial identification of the gaps through ImageJ software by Fiji [83] and the consequent analysis of their extension through a customised MATLAB (Mathworks, Natick, MA, USA) code, to calculate the area (both total and as a frequency distribution) of the opened gaps.

As a first step, all the images taken for each vascular channel were stitched together in a mosaic of tiles giving the overview of the conditions of the entire endothelial layer in the microchannel. This was done using the ImageJ Grid/Collection Stitching plugin [83,84], which aligns consecutive frames, recognising their overlapping areas, and thus checking their position in the image.

**Figure 5:**
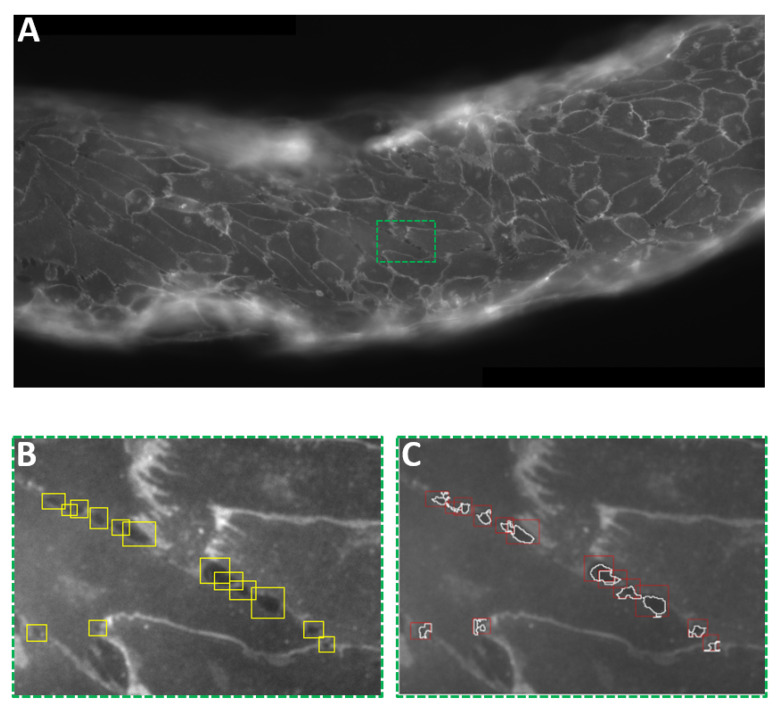
Interendothelial gap analysis. (**A**) Portion of the stitched confocal fluorescence image of the vascular channel. (**B**) Cropped image from (**A**). Interendothelial gaps opened upon cavitation are manually selected as rectangular ROIs in ImageJ. A list of ROIs is thus obtained and processed as described in Section 2.9. (**C**) Same cropped image as in (**B**), after processing. The code operates directly on the ROIs selected in the TIF file, identifying the gap contained in each rectangular ROI and its boundaries.

The global image simplifies visualisation of the interendothelial gaps, as in the example shown in Figure 5A. As a preliminary step, rectangular regions of interest (ROIs) centred on a single gap were manually selected using the ImageJ interface, Figure 5B. As an output, the selected ROIs are listed in a TXT file containing information about their coordinates, width and height. The original TIF (stitched) image together with the TXT file carrying information on the ROIs are thus processed with the customised image analysis code.

Reading through the list of ROIs, the program crops each previously selected region. The cropped images are equalised to improve contrast. Successively, they undergo binarisation based on a thresholding method, whereby all the ROI pixels above a specific cut-off value are identified as “signal”, i.e., belonging to the interendothelial gaps. Within each ROI, connected pixels above the threshold are considered part of a blob. Since, sometimes, more than a single blob is found in one ROI, selection is performed on the basis of distance from the centre of mass: leveraging on the effectiveness of the manual selection procedure, aimed at isolating single gaps, the program computes the centre of mass of each blob and its distance from the ROI centre. The blob closest to the centre is eventually identified as the interendothelial gap, see Figure 5C, to be quantified in terms of area, perimeter and other geometrical properties.

## 3. Results and Discussion

**Figure 6:**
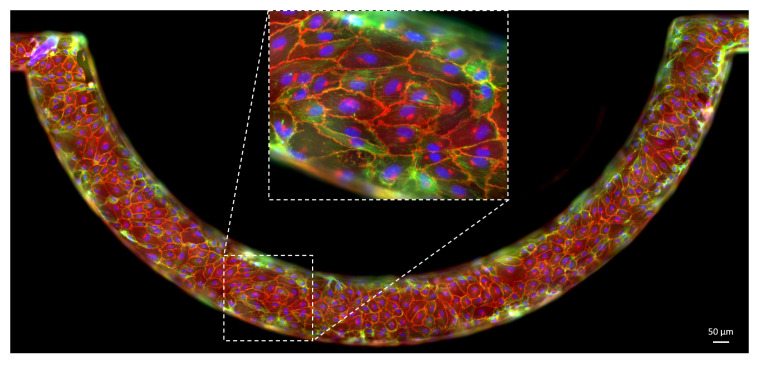
Fluorescence image of an endothelial monolayer lining the walls of the vascular channel. The endothelium shows full-maturation features, with VE-cadherin (red) arranged in a continuous linear pattern in the interendothelial junctions. Actin filaments (green) form stress fibres or cortical filaments interacting with junctional complexes and driving their organisation. Cell nuclei are stained with DAPI (blue). The inset highlights the characteristics of the mature endothelial barrier. The blurry regions at the vascular channel edges are due to the three-dimensional structure of the endothelium. They are the effect of the superimposition of fluorescence from layers at different depth along the lateral walls of the microchannel.

Immunolabeling of actin filaments and VE-cadherin allowed to assess the constant reorganisation of cell cytoskeleton and junctional complexes. Initially, a copious amount of actin stress fibres across the cytoplasm were recorded, associated to a discontinuous VE-cadherin pattern mediating the preliminary cell–cell contacts. Upon endothelial maturation, actin cytoskeleton showed a well-organised cortical network, driving VE-cadherin clusterisation at interendothelial junction sites, in a linear uninterrupted pattern as in Figure 6. These characteristics denote the establishment of a confluent endothelial resting state. These processes were also accompanied by the tightening of the cell layer and the acquisition of a mature compact cobblestone cell phenotype [85], with a progressive reduction of cell perimeter and the acquisition of a polygonal shape. Moreover, streamwise cell elongation was observed.

**Figure 7:**
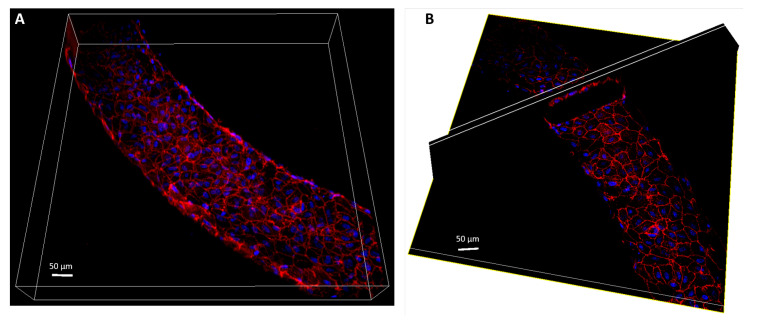
Three-dimensional reconstruction of the endothelium based on a confocal Z-stack acquisition where VE-cadherin (red) and cell nuclei (blue) are stained. (**A**) Three-dimensional rendering of the vascular channel. (**B**) Three-dimensional orthogonal view of the endothelium to obtain a compound figure showing the horizontal section combined with a diagonal cross section.

Figure 7 shows the three dimensional reconstruction of the endothelium in the microfluidic platform, see also the video provided as Supplementary Material. The image is constructed based on the Z-stack confocal acquisition of the endothelium where nuclei (blue) and VE-cadherin (red) were stained. The cross section shows that the endothelial monolayer adheres to the PDMS walls of the microchannel. Cells organise in a continuous integer barrier around the whole vascular microchannel, resembling vessels. The experimental set-up provides cells with the same mechanical stimuli and shear stress levels as in human microvasculature, and promotes the formation of a mature functional endothelium. Indeed, shear stress crucially affects ECs phenotype, including the efficiency of cell adhesion to the substrate (focal adhesions), the strength of interendothelial junction complexes, formed by VE-cadherin, as well as the organisation of actin cytoskeleton [33,34,35,86]. In this context, the chosen flow rate values were proven to be optimal for the physiological maturation of the endothelial barrier. Indeed, flow rate increase causes cell detachment from the vessel walls. Analogously, too low flow rate does not allow proper ECs organisation.

**Figure 8:**
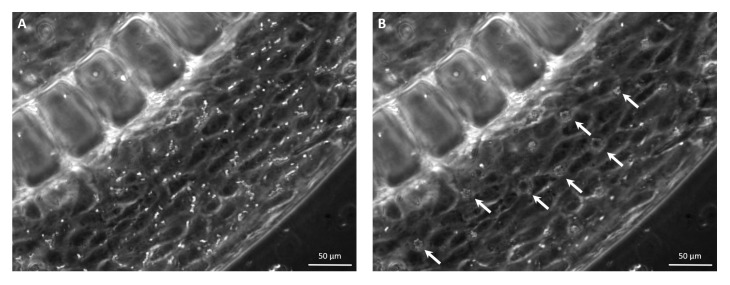
Phase contrast images showing the events related to US exposure in the blood vessel model. The images show a portion of the vascular channel, where MBs have been injected. (**A**) Prior to irradiation, MBs appear as homogeneously-distributed, dispersed particles. (**B**) Upon US exposure, MBs respond to the acoustic pressure by aggregating and forming evenly-spaced clusters (white arrows).

Once injected into the vascular channel, MBs were observed to distribute homogeneously along the channel and to have a tendency to near the upper wall of the microchannel, due to buoyancy. Upon the forces exerted by the US beam, MBs slow down and tend to cluster, forming evenly-spaced agglomerates, as shown in Figure 8. Upon MBs exposure to US, cavitation occurs, exerting mechanical stimuli that eventually increase the permeability of the endothelial barrier.

The insonation parameters were chosen within the ranges recommended for protocols for medical applications [87]. In this regard, the US irradiation frequency should range between 0.3 and 3MHz, the intensity between 0.3 and $3\;W\;{\mathrm{cm}}^{-2}$. These parameters account for US penetration depth and spatial resolution, and for temperature increase in the tissues, respectively. According to safety standards, the two other crucial parameters are the mechanical index (MI) and the thermal index (TI). The MI accounts for US-mediated mechanical effects (i.e., cavitation) and is defined as the ratio of US peak negative pressure (MPa) to centre frequency square root (MHz), $\mathrm{MI}=p_{min}/\sqrt{f}$. Analogously, TI is an indicator for temperature increase and is given by the ratio of the relevant acoustic power at the target tissue to the power needed to rise tissue temperature by 1 ${}^{\circ }$C, $\mathrm{TI}=W_{p}/W_{deg}$. Values of MI (MPa/MHz) and TI lower than “1” are generally considered safe, whereas the MI value of 1.9 MPa/MHz is set by FDA as the upper safety limit [50,87,88]. The transducer drivings, 80mV and 140mV, correspond to $5\;{W\;\mathrm{cm}}^{-2}$ and $17\;{W\;\mathrm{cm}}^{-2}$ irradiation intensity, respectively, conforming to clinical standards. The use of MBs, that act as acoustic amplifiers, allow to reduce the overall US exposure, thus decreasing the mechanical stress exerted on the vasculature. Among other possibilities, SonoVue^®^ were chosen for their exhaustive characterisation [77] and their common employment in diagnostics (EMA).

**Figure 9:**
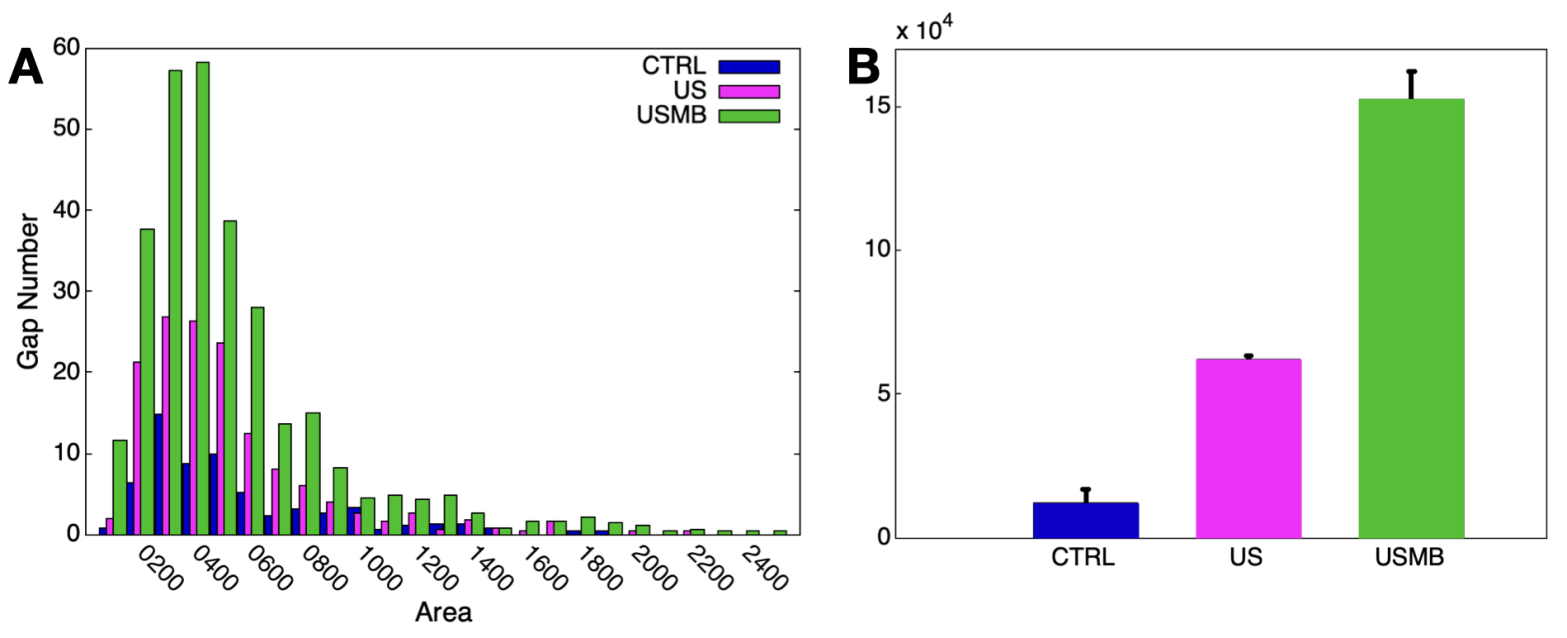
Histograms of gap area frequency distribution (**A**) and total gap area (**B**) for each tested condition (control, US alone and USMB).

A crucial aspect is the individuation of a quantitive observable to measure the USMB exposure effects on vascular permeability. As discussed in [35], a suitable parameter is the area of the interendothelial gaps opened on account of cavitation. The major output of the analysis consists of the frequency distribution of gap area, as shown in Figure 9A, and of the total area opened by the cavitation process, Figure 9B. The histograms show outstanding effects of the endothelium exposure to US in the presence of MBs at an acoustic pressure of 0.72 MPa, which is found particularly effective in enhancing the barrier permeability [35]. A substantial increase in the number of gaps opened between adjacent cells has been registered both for USMB and US alone conditions with respect to the control (no exposure). Notwithstanding, the extent of this phenomenon is significantly wider in USMB condition, as shown by the bars referred to the total gap area, which indicate, with respect to control, an increase of 130% for endothelial exposure to US alone and up to the 360% for USMB condition. It has been shown [35] that, once the irradiation ceases, the endothelium restores its original integrity, with total gap closure and recover of the barrier functionality, demonstrating that the applied protocol show potential for a safe application in clinics.

## 4. Conclusions and Outlook

The vasculature-on-chip we described provides a reproducible and well-defined experimental model that resembles human vessel physiology in an in vitro micrometre-sized device, paving the way for a wide range of human-focused investigations where the experimental conditions can be finely tuned. The model was focused on the endothelial layer constituting the inner part of blood vessels. The combination of cell biology and microfluidics allows to recreate a physiological-like microenvironment, where ECs can be cultured under flow conditions in a 3D fashion. The possibility to control the shear stress is found crucial for the maturation of the endothelium, leading to the formation of a compact monolayer of cells lining the channel walls and promoting their adhesion to the substrate.

The developed system was employed to investigate an enhanced drug delivery strategy involving US and the same MBs (SonoVue^®^) already employed in clinics and diagnostics. The presence of MBs amplifies the effect of US irradiation, leading to the reversible opening of intercellular gaps temporarily increasing the permeability of the endothelium.

Image analysis allowed to identify the gaps as blobs within the selected ROIs providing data for gap area statistics (area distribution, total area, shape factor) and allowing us to compare the different experimental conditions.

The proposed procedure lays the groundwork for further experimental work aimed at shedding light on elusive aspects of the process, such as interendothelial gap dynamics. Indeed, the time frame over which these events occur is still a matter of controversy. The opening of interendothelial gaps is known to be followed by complete reversion of the phenomenon within 45–60 min after insonation [35,60]. There are, however, indications that, at the cell membrane level, sonoporation events recover within a milliseconds-to-minutes time range [89,90]. Concerning the endothelial tissue and cell junctions, the relative information is still missing. Our experimental protocol offers the chance of real-time observation of gap dynamics and, thanks to the versatility of the microfluidic platform, permits us to realise different experimental conditions, physiological and pathological, as well as to address a plethora of pharmaceutical treatments that can be administered through functionalised MBs.

The microvasculature model can be further developed under several aspects. Concerning image analysis, at the present stage, gap-containing ROIs are still selected manually. In view of more massive experimental campaigns, the development of an automatic detection algorithm for interendothelial gap identification would greatly speed up the data analysis phase. The micro-bio-system is extremely flexible, allowing to increase the physiological complexity of the model in a relatively simple way to better reproduce the vascular microenvironment. This flexibility can be exploited, in particular, to investigate pathological conditions aimed at addressing, e.g., the effect of cancer and its microenvironment on the endothelium. At the present stage, the microfluidic set up does allow only the indirect evaluation of the barrier permeability by fluorescent dye diffusion from the microvessel into the inner tissue chamber. However, the chip design can be modified to include electrodes for on-board electrokinetic measurements of barrier permeability that would be crucial for real time evaluation of USMB-induced permeability enhancement.

In conclusion, the multi-step protocol we have developed can be exploited for a range of studies concerning the response of the endothelium to US-induced cavitation in presence of different complementary stimuli, purely mechanical, chemical and physiological, providing the chance to bridge the gap between traditional in vitro (2D cell cultures) and studies.

## Supplementary Materials

The following are available online at https://www.mdpi.com/article/10.3390/mi12060658/s1. Additional detail and a video showing the 3D structure of the endothelium.
