# Supplementary material for: A Microfluidic Platform for Cavitation-Enhanced Drug Delivery
Source: Micromachines (Basel). 2021 Jun 3;12(6):658. doi: 10.3390/mi12060658 (PMC8229805; doi:10.3390/mi12060658)
Supplement: Supplementary file 1 [file micromachines-12-00658-s001.zip › Micromachines_2021_SM.pdf]

Supplementary Material to the paper

# A microfluidic platform for cavitation-enhanced drug delivery

by

G. Grisanti, D. Caprini, G. Sinibaldi, C. Scognamiglio, G. Silvani, G. Peruzzi & C.M. Casciola

**- Configuration of the insonation chamber**

Figure 1S shows a picture of the insonation chamber installed on the microscope stage.

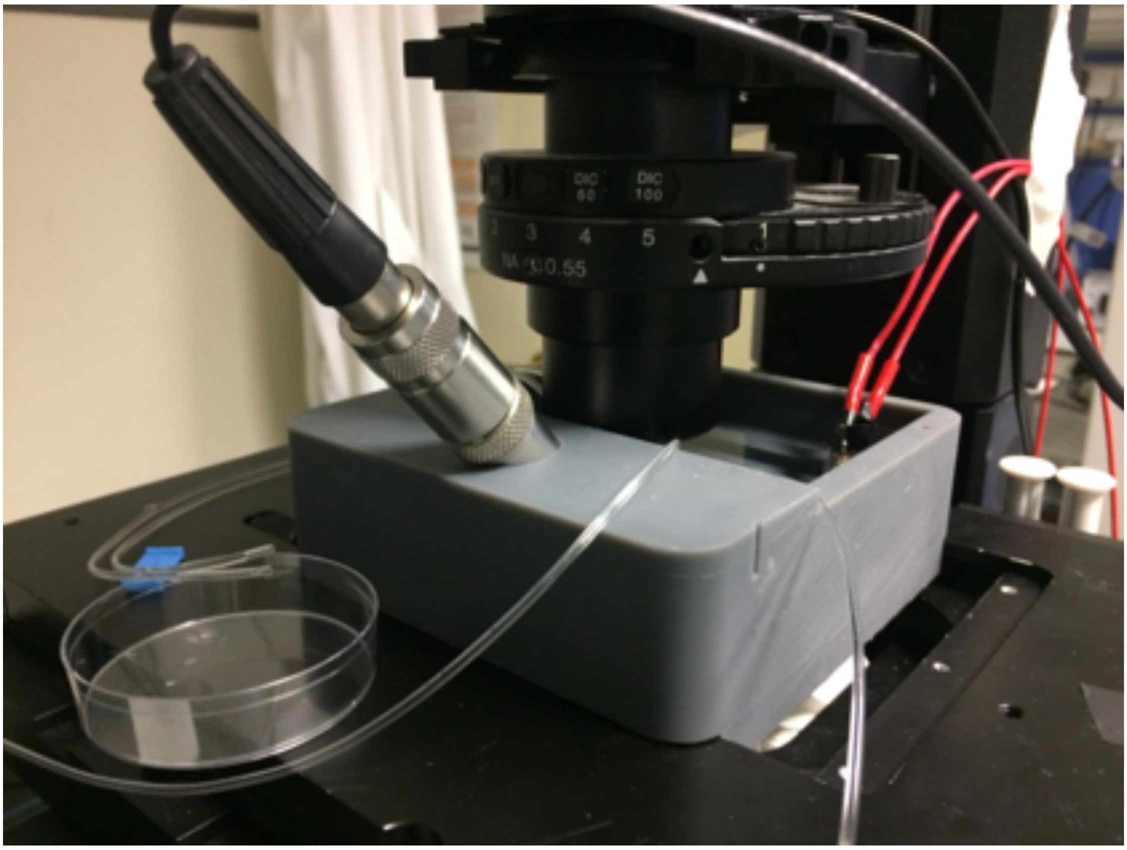


Figure 1S. Arrangement of the insonation chamber hosting the acoustic piezo on the microscope stage. The chamber hosts the microfluidic device at its bottom, guaranteeing optical access to the microscope objectives. In order to allow US waves to correctly propagate from the $45^0$-inclined piezo located at the top, the chamber was filled with deionised water. Water temperature was constantly monitored and maintained at $37^0$C with the aid of a thermal controller. The piezo was connected to the US chain, comprising signal generator, 50-dB amplifier, and oscilloscope to monitor the US bursts.

- **Transducer calibration**

Preliminarily to cavitation experiment, the transducer was calibrated using a needle hydrophone, for both transducer driving voltages of 80 mV and 140 mV, as shown in Fig. 2S.A.

The X-Y plane was scanned in order to find the coordinates of the maximum acoustic pressure. Once found this point, the Z axial direction was scanned to determine near and far fields of the emitter. The distance Z ≈ 25 mm was identified as the boundary between near and far fields, as indicated in Fig. 2S.C.

This characterisation was congenial to the design of the insonation chamber. Indeed, as already explained, the transducer was mounted onto the specific support in the insonation chamber so that the US beam is directed toward the centre of the microfluidic chip, placed at the bottom of the chamber. The transducer inclination and its distance from the device were designed to be $45^0$ and 35 mm, respectively, in order to guarantee US irradiation in the far field, thus preventing uncontrolled pressure variations.

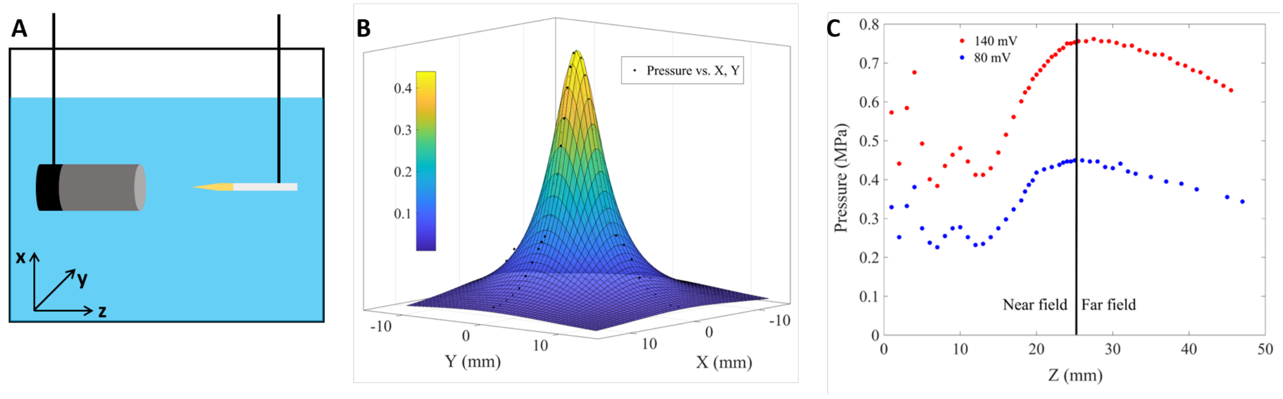


Figure 2S. **A.** Sketch of the calibration setup, carried out in a plexiglass box filled with deionised water at RT. **B.** Graph showing pressure distribution at 80 mV driving voltage in a X-Y cross plane at the axial position Z = 30 mm. **C.**Plot depicting the maximum cross-plane acoustic pressure for each axial distance Z from the emitter, for both 80 mV (blue) and 140 mV (red) driving voltage. The solid line at Z = 25 mm marks the boundary between acoustic near and far fields.

- **Supplementary Movie**

The Video shows the 3D rendering of the endothelium as reconstructed from Z-stack images acquired at the confocal microscope. The structure is viewed from different angles to provide a visual impression of the three dimensional structure of the blood vessel on a chip.
